# Modulation of IMD, Toll, and Jak/STAT Immune Pathways Genes in the Fat Body of *Rhodnius prolixus* During *Trypanosoma rangeli* Infection

**DOI:** 10.3389/fcimb.2020.598526

**Published:** 2021-01-18

**Authors:** Agustín Rolandelli, Adeisa E. C. Nascimento, Leticia S. Silva, Rolando Rivera-Pomar, Alessandra A. Guarneri

**Affiliations:** ^1^ Centro de Bioinvestigaciones (CeBio), Centro de Investigaciones y Transferencia del Noroeste de la Provincia de Buenos Aires (CIT-NOBA), Universidad Nacional del Noroeste de la Provincia de Buenos Aires (UNNOBA)—Consejo Nacional de Investigaciones Científicas y Técnicas (CONICET), Pergamino, Argentina; ^2^ Vector Behaviour and Pathogen Interaction Group, Instituto René Rachou, Fundação Oswaldo Cruz (FIOCRUZ), Belo Horizonte, Brazil

**Keywords:** trypanosomatids, vector-parasite interactions, immune response, parasite load, kissing bugs

## Abstract

*Trypanosoma rangeli* is the second most common American trypanosome that infects man. It is vectored by triatomines from the genus *Rhodnius*, in which it invades the hemolymph and infects the salivary glands, avoiding the bug immune responses. In insects, these responses are initiated by well conserved pathways, mainly the IMD, Toll, and Jak/STAT. We hypothesize that long-term infection with *T. rangeli* in the gut or hemolymph of *Rhodnius prolixus* triggers different systemic immune responses, which influence the number of parasites that survive inside the vector. Thus, we investigated groups of insects with infections in the gut and/or hemolymph, and evaluated the parasite load and the expression in the fat body of transcription factors (*Rp-Relish*, *Rp-Dorsal*, and *Rp-STAT*) and inhibitors (*Rp-Cactus* and *Rp-Caspar*) of the IMD, Toll, and Jak/STAT pathways. We detected lower parasite counts in the gut of insects without hemolymph infection, compared to hemolymph-infected groups. Besides, we measured higher parasite numbers in the gut of bugs that were first inoculated with *T. rangeli* and then fed on infected mice, compared with control insects, indicating that hemolymph infection increases parasite numbers in the gut. Interestingly, we observed that genes from the three immune pathways where differentially modulated, depending on the region parasites were present, as we found (1) *Rp-Relish* downregulated in gut-and/or-hemolymph-infected insects, compared with controls; (2) *Rp-Cactus* upregulated in gut-infected insect, compared with controls and gut-and-hemolymph-infected groups; and (3) *Rp-STAT* downregulated in all groups of hemolymph-infected insects. Finally, we uncovered negative correlations between parasite loads in the gut and *Rp-Relish* and *Rp-Cactus* expression, and between parasite counts in the hemolymph and *Rp-Relish* levels, suggesting an association between parasite numbers and the IMD and Toll pathways. Overall, our findings reveal new players in *R. prolixus*–*T. rangeli* interactions that could be key for the capacity of the bug to transmit the pathogen.

## Introduction

*Trypanosoma rangeli* is a protozoan parasite vectored by triatomine bugs, especially those from the genus *Rhodnius*, and is able to infect humans and other mammals. However, in contrast to *Trypanosoma cruzi*, the etiologic agent of Chagas disease, *T. rangeli* is considered unable to elicit pathology in mammals, though it is detrimental to its insect vector (Watkins, 1971; Guarneri and Lorenzo, 2017). In addition, both parasites share biological characteristics, antigens, geographical distribution, as well as insect and vertebrate hosts, which can compromise the correct diagnosis of *T. cruzi* infection due to crossed serological reactions (Vieira C. B. et al., 2018).

*Rhodnius prolixus* is considered one of the most efficient *T. cruzi* and *T. rangeli* vector in South America. It is a classical model organism in which basic concepts of insect physiology were initially established by Professor Sir Vincent B. Wigglesworth, with a relatively rapid life cycle and easy to breed in the laboratory, compared with other triatomine species (Vieira et al., 2016; Azambuja et al., 2017). In addition, *R. prolixus* is the only triatomine with its full genome sequenced to date, making it an ideal model to investigate arthropod-parasite interactions in a non-dipteran blood feeding insect vector (Mesquita et al., 2015).

The interactions between *T. rangeli* and *R. prolixus* begin when parasites are ingested as blood trypomastigotes, when the triatomine feeds on infected hosts (Hecker et al., 1990). After ingestion, the parasites transform into epimastigotes in the digestive tract and eventually cross the intestinal epithelium, reaching the hemocoel and hemolymph. Finally, *T. rangeli* invades and multiplies in the salivary glands, where it differentiates into metacyclic trypomastigotes, which are the infective forms transmitted during blood-meal to new vertebrate hosts (Guarneri and Lorenzo, 2017).

In order to establish the infection, *T. rangeli* must overcome the barriers imposed by the triatomine immune response in both the digestive tract and hemocoel. In insects, this response is initiated by host pattern receptor proteins (PRPs), which recognize pathogen associated molecular patterns (PAMPs) on the surface of pathogens and elicit different well conserved immune pathways: the immune deficiency (IMD), the Toll and the Janus kinase/Signal Transducers, and Activators of Transcription (Jak/STAT) (Salcedo-Porras and Lowenberger, 2019). Apparently, each of them acts in different types of infection, and induces the expression of certain subset of antimicrobial peptides (AMPs): Gram-negative bacteria trigger the IMD pathway, Gram-positive bacteria and fungi initiate the Toll pathway, and viruses activate the Jak/STAT pathway (Salcedo-Porras et al., 2019). Nevertheless, studies in ticks, lice, and hemipterans point to a deviation from the paradigm of a complete conservation of immune pathways in arthropods, emphasizing the need to investigate immune responses in different subphyla (Kim et al., 2011; Palmer and Jiggins, 2015; Shaw et al., 2018; Nishide et al., 2019).

Most of the key molecules for the IMD, Toll, and Jak/STAT pathways are present in the genome and many transcriptomes of *R. prolixus* (Ribeiro et al., 2014; Mesquita et al., 2015; Ons et al., 2016; Latorre-Estivalis et al., 2017; Zumaya-Estrada et al., 2018). The gene encoding the protein *Relish* (*Rp-Relish*), a conserved transcription factor (TF) related to the IMD pathway, was identified in *R. prolixus* genome; while its inhibitory protein *Caspar* (*Rp-Caspar*) was not detected until a recent deeper analysis using BLAST complemented with hidden Markov model profiles (Mesquita et al., 2015; Salcedo-Porras et al., 2019). Moreover, the coding sequences for the NF-κB TF *Dorsal* (*Rp-Dorsal*) and its inhibitor protein *Cactus* (*Rp-Cactus*) of the Toll pathway were detected in *R. prolixus* by suppressive subtractive hybridization technique and by transcriptome analysis, respectively (Ursic-Bedoya and Lowenberger, 2007; Ursic-Bedoya et al., 2009; Ribeiro et al., 2014). In addition, a transcript coding for the TF *STAT* (*Rp-STAT*) of the Jak/STAT pathway was also observed in transcriptomes from the *R. prolixus* digestive tract (Ribeiro et al., 2014). Furthermore, AMPs, such as lysozymes, defensins and prolixicin, have also been isolated from *R. prolixus* digestive tract and fat body (Lopez et al., 2003; Ursic-Bedoya et al., 2008; Ursic-Bedoya et al., 2011).

AMPs are mainly synthesized by fat body cells, which release them into the hemolymph for systemic delivery; or by epithelial cells for local secretion (Vieira et al., 2016; Azambuja et al., 2017; Salcedo-Porras and Lowenberger, 2019). Remarkably, there are several reports that investigate the induction of AMPs, which are the main effector molecules of the IMD, Toll, and Jak/STAT immune pathways, in *R. prolixus* challenged with different microorganisms (Castro et al., 2012; Vieira et al., 2014; Mesquita et al., 2015; Vieira et al., 2015; Vieira et al., 2016; Azambuja et al., 2017; Salcedo-Porras et al., 2019). Nevertheless, there are very few studies that investigate the immune pathways triggered by pathogens that lead to the production of those AMPs, or whether there are differences in the pathways activated according to the type, route and duration of infection. In this regard, *Rp-Relish* was found to regulate certain AMPs in response to Gram-negative and Gram-positive bacteria, in the fat body of *R. prolixus* (Salcedo-Porras et al., 2019). Furthermore, other report demonstrated that *Rp-Relish* controls defensins and lysozymes expression; and its repression led to an increase in the population of the symbiotic bacteria *Rhodococcus rhodnii*. Strikingly, the same study revealed that silencing *Rp-Relish* or *Rp-Dorsal* did not influence in *T. cruzi* loads detected 1 or 2 weeks after the infection (Mesquita et al., 2015). However, in a recent report, a reduction in *T. cruzi* loads was evidenced in the midgut of *R. prolixus* treated with the drug IMD-0354, which blocks IκBα phosphorylation and thus prevents nuclear translocation of NF-κB TFs (*Rp-Relish* and *Rp-Dorsal*), indicating the importance of these pathways in the anti-parasitic innate immune responses (Vieira C. S. et al., 2018).

Several studies demonstrate that oral infection with *T. rangeli* modulates systemic immune responses in *R. prolixus*, even without (or before) the penetration of parasites into the hemolymph (Gomes et al., 2003; Garcia et al., 2004b; Whitten et al., 2007; Figueiredo et al., 2008). In addition to *T. rangeli*, *T. cruzi* (which do not infect the hemolymph) also modulates the systemic immune responses when infecting the digestive tract (Vieira et al., 2016; Favila-Ruiz et al., 2018). Furthermore, the growth of the native microbiota in the gut following a blood meal elicits the up-regulation of *Rp-Relish* in the fat body (Mesquita et al., 2015). These evidences support a significant role of systemic immune responses in controlling localized microbial infection. Accordingly, in this work we measured the expression of TFs (*Rp-Relish*, *Rp-Dorsal*, and *Rp-STAT*) and inhibitors (*Rp-Cactus* and *Rp-Caspar*) of the IMD, Toll, and Jak/STAT immune pathways in the triatomine’s fat body, the main organ that synthesize and release in the hemolymph inducible humoral immune factors to establish a systemic response. Furthermore, we analyzed parasite loads in the gut and hemolymph of *R. prolixus* with different forms of infection with *T. rangeli*, and we evaluated whether there was a correlation between the parasite counts and the expression of these immune genes. We hypothesize that long-term infection with *T. rangeli* in the digestive tract or hemolymph of *R. prolixus* triggers different systemic immune responses, which influences the number of parasites that survive in both regions of the insect body.

## Materials and Methods

### Insect Rearing

*Rhodnius prolixus* were obtained from a colony maintained by the Vector Behavior and Pathogen Interaction Group at René Rachou Institute (FIOCRUZ, Minas, Brazil), which was established from insects collected in Honduras around 1990. The colony was maintained under controlled temperature (26 ± 1°C), relative humidity (65 ± 10%), and natural illumination cycle. Insects were consistently fed on diverse sources of blood that included mice, chicken, and a membrane feeder offering citrated rabbit blood at 37°C. Mice and chickens were anesthetized with intraperitoneal injections of ketamine (150 mg/kg; Cristália, Brazil) plus xylazine (10 mg/kg; Bayer, Brazil), and ketamine (20 mg/kg; Cristália, Brazil) plus detomidine (0.3 mg/kg; Syntec, Brazil), respectively. *Rhodnius prolixus* 4^th^ instar nymphs starved for 30 days were used in all experiments.

### *Trypanosoma rangeli* Culture

*Trypanosoma rangeli* CHOACHI strain used in this study was originally isolated from naturally infected *R. prolixus* (Schottelius, 1987). The epimastigote forms used to infect insects were cultured by twice weekly passages at 27°C in liver-infusion tryptose (LIT) medium, supplemented with fetal bovine serum 15%, 100 units/ml penicillin, and 100 μg/ml streptomycin. In order to maintain the strain infectivity, parasites were passed through triatomines and mice every 3 months (Rodrigues et al., 2016).

### Insect Infections

Insects were infected as recently described (Guarneri, 2020). For oral infection, sixty-day old male *Swiss Webster* mice were used. *Trypanosoma rangeli*-infected mice were generated through the bite of 5^th^ instar nymphs containing trypomastigote forms in their salivary glands and were used at 14 days post infection (pi). Mice parasitemia was confirmed immediately before starting the experiments (2.5–5 × 10^3^ parasites/ml).

For intracoelomic inoculation, *T. rangeli* epimastigotes were obtained from 10 day cultures, and resuspended in sterile PBS (0.15 M NaCl in 0.01 M sodium phosphate, pH 7.4) in a concentration of 5 × 10^4^ parasites/ml. *Rhodnius prolixus* nymphs were inoculated through the thoracic pleura with 1 μl of PBS + LIT or PBS + the parasite suspension, using a 50 μl syringe (Hamilton Company, NV, USA, needle 13 × 3.3; ½”) connected to a dispenser (model 705, Hamilton Company, NV, USA).

#### Experimental Design 1

To simulate a natural infection with the parasite, *R. prolixus* nymphs were randomly separated and fed on healthy or *T. rangeli*-infected mice (oral infection). Each insect was allowed to ingest 20–30 μl of blood, which resulted in ingestion of approximately 50–150 trypomastigotes. A week later, both groups of insects were randomly divided and inoculated in the hemocoel with 1 μl of PBS + LIT or PBS + parasite suspension (5×10^4^ epimastigotes/ml). The inoculation was performed since *T. rangeli* does not always invade the hemocoel in the natural course of the insect infection (Ferreira et al., 2010). One week after inoculation (2 weeks after oral infection), hemolymph samples were taken by cutting off the terminal segment of a hind leg and collecting a drop of hemolymph on a microscope slide to check the status of infection. Thus, five experimental groups representing different forms of *T. rangeli* infection were generated: (1) insects without parasites in gut or hemolymph (G−H−, control group); (2) insects with parasites in the gut, but not in the hemolymph (G+H−); (3) insects that were only infected by inoculation in the hemolymph (G−H+); (4) insects with parasites in the gut and further inoculated with parasites in the hemolymph (G+H+); and (5) insects that were only gut-infected, but with parasites in hemolymph by “natural” crossing from the intestinal lumen (G+H+Nat). Two weeks after the inoculation all groups of insects were fed on healthy mice. Finally, a week later (4 weeks since the beginning of the experiment; 3 weeks after inoculations) parasite counts and gene expression were determined in individual insects.

The experimental design was repeated three times (three biological replicates), and insects used in subsequent experiments came from all the three biological replicates (n = 26–28 insects per group for parasite counts, with 8–10 insects from each replicate; and n = 8–10 insects per group for gene expression, with 2–4 insects from each replicate), except for the G+H+Nat group that only appeared in two of the replicates (n = 12 insects for parasite counts, with eight and four insects from each replicate; and n = 8 insects for gene expression, with four insects from each replicate).

#### Experimental Design 2

This design was only used to determine whether hemolymph infection could alter the parasite load in the gut of *R. prolixus* orally infected with *T. rangeli*. For this reason, the experiment was performed based on the first experimental design, but insects were first inoculated in the hemocoel and then orally infected. Accordingly, *R. prolixus* nymphs were fed on healthy mice. A week later, insects were randomly divided and inoculated with 1 μl of PBS + parasite suspension containing 50 or 500 parasites, or the same volume of PBS + LIT. Thus, four experimental groups were obtained: insects infected by inoculation with PBS + 50 epimastigotes (H+50), insects infected by inoculation with PBS + 500 epimastigotes (H+500), and their respective PBS + LIT inoculated control groups (H−50 and H−500, without hemolymph infection). Two weeks after the inoculation, all groups of insects were orally infected by feeding 20–30 μl of blood on *T. rangeli*-infected mice. One week later (4 weeks since the beginning of the experiment) parasite counts were determined. Two biological replicates were performed for this experiment, and 4–5 insects per group from each replicate were used for parasite counts.

After the inoculation, the survival of the insect was >95% in each of the experimental group, in all biological replicates, in both experimental designs.

### Quantification of Parasite Loads

#### Parasite Loads in the Hemolymph

2 μl of hemolymph samples from individual insects were taken by cutting off the terminal segment of the right hind leg and gently pressing the abdomen. Samples were immediately diluted in 18 μl of anticoagulant solution (0.01 M ethylenediamine tetra-acetic acid, 0.1 M glucose, 0.062 M sodium chloride, 0.03 M trisodium citrate, 0.026 M citric acid, pH 4.6), in a proportion of 1:10 hemolymph:anticoagulant (Mello et al., 1995). Parasite loads were estimated by directly counting in a Neubauer chamber.

#### Parasite Loads in the Gut

After hemolymph extraction, the cuticle of the insects was cut laterally and the entire digestive tract (anterior midgut, posterior midgut and rectum) was carefully removed with tweezers under a stereo microscope. Each digestive tract was washed three times in PBS and then transferred to a 0.5 ml plastic tube, where it was homogenized in 20 μl of PBS. Afterward, parasite loads were estimated by directly counting in a Neubauer chamber.

### Fat Body Isolation, RNA Extraction, and cDNA Synthesis

After gut removal, the thin layer of abdominal fat body beneath the dorsal and ventral cuticle was dissected and immediately transferred to a 1.5-ml tube containing 1 ml of TRizol reagent (Thermo Fisher Scientific, MA, USA). Fat body RNA was extracted from individual insects following the manufacturer’s recommendations, concentrations were determined using a Qubit 2.0 Fluorometer (Thermo Fisher Scientific, MA, USA), and RNA integrity was checked by agarose gel electrophoresis. Afterward, DNAse treatment was carried out in 20-μl reactions with RNase-free DNase I (TransGen Biotech, Beijing, China) for 15 min at 37°C, using 1 μg of total RNA. Then, cDNA synthesis was performed in 20-μl reactions containing 500 ng of DNAse treated RNA using the EasyScript Reverse Transcriptase (TransGen Biotech, Beijing, China) with anchored Oligo(dT)_18_ Primer, following the manufacturer’s instructions. The subsequent cDNA was diluted 1:2 with DEPC water.

### Real-Time Quantitative Polymerase Chain Reaction

Real-time quantitative polymerase chain reactions (RT-qPCR) were conducted using an Applied Biosystems 7500 Real-Time PCR System (Thermo Fisher Scientific, Waltham, MA, USA). Primers were designed for: *α-Actin* (housekeeping), *Rp-Relish*, *Rp-Dorsal*, *Rp-Caspar*, and *Rp-STAT*. Primers for *Rp-Cactus* were retrieved from the literature (Vieira C. S. et al., 2018). RT-qPCR efficiency for each primer pair was determined using the slope of a linear regression mode. The sequences, amplicon length and RT-qPCR efficiency of each of the primer pair are shown in **Table 1**. The identity of each of the amplicons was confirmed by Sanger sequencing (Standard-Seq, Macrogen, Seoul, Rep. of Korea).

**Table 1:** Primer sequences, amplicon lengths, and RT-qPCR efficiencies.

Gene	Primer sequence (5′ to 3′)	Amplicon length (bp)	RT-qPCR efficiency (%)
*α-Actin*	Fw-GACTTGGCTGGTCGTGATCT	201	95.81%
	Rv-ACCATCAGGCAATTCGTAGG		
*Rp-Relish*	Fw-CCGCATGGACCATTAACTG	167	108.55%
	Rv-AGGTTCACTTCTTACACTTCTTC		
*Rp-Caspar*	Fw-GATGGTAGTGTATTGACGAATG	196	106.28%
	Rv-CGGTGACAGTGATCTTGC		
*Rp-Dorsal*	Fw-GTCATTGTTCTGGTTCAGTAGC	181	91.25%
	Rv-GGCGTTCGGAATGAAATAGC		
*Rp-Cactus*	Fw-GTGCTGGTGCTTGTACGAAA	154	96.84%
	Rv-GGAGTCGGACGATACCTCAA		
*Rp-STAT*	Fw-GGCACCGAATCAGTAATGG	173	94.17%
	Rv-AAAGCATTATCCCAGGTTACC		

All RT-qPCR reactions contained 1 μl (~12.5 ng) of cDNA, 0.3 μl of each primer (10 mM), 5 μl of iTaq Universal SYBR Green Supermix (Bio-Rad Laboratories, CA, USA), and 3.4 μl of DEPC water in a final volume of 10 μl. The RT-qPCR conditions used were: 95°C for 5 min; 35 cycles of: 95°C for 15 s, 55°C for 30 s and 72°C for 30 s; followed by a melting curve analysis to confirm the specificity of the reaction. No-template controls and no-RT controls were included to verify the absence of exogenous DNA, primer-dimers formation and/or contamination. Reactions on each sample and controls were run in duplicate. Relative differences in transcripts levels were calculated considering the amplification efficiency of each primer pair using the Pfaffl method (Pfaffl, 2001), with *α-Actin* as reference gene. All data were normalized relative to values recorded for non-infected control insects (G−H− group).

### Statistical Analysis

Shapiro-Wilk normality test was applied for assessing normality in the data set. The non-parametric Mann-Whitney U test was used for comparison between two experimental groups. One-way ANOVA followed by Bonferroni multiple-comparison *post-hoc* test was performed to analyze differences between three or more experimental groups. Correlation analyses were carried out using the Spearman correlation test. P values of < 0.05 were considered statistically significant.

## Results

### *T. rangeli* Numbers in the Gut of *R. prolixus* Are Higher When the Hemolymph Is Infected

First, we investigated whether there were differences in parasite loads in the gut or hemolymph of *R. prolixus* with different forms of infection with *T. rangeli*. As detailed in Methods section (Experimental design 1), we analyzed five different groups of long-term infected insects: (1) without gut or hemolymph infection (G−H−); (2) without parasites in the gut, but infected in the hemolymph by inoculation (G−H+); (3) with parasites in the gut, but not in the hemolymph (G+H−); (4) with parasites in the gut and further inoculated with parasites in the hemolymph (G+H+); and (5) with parasites in the gut and in the hemolymph, but by “natural” crossing from the intestinal lumen (G+H+Nat).

**Figure 1:**
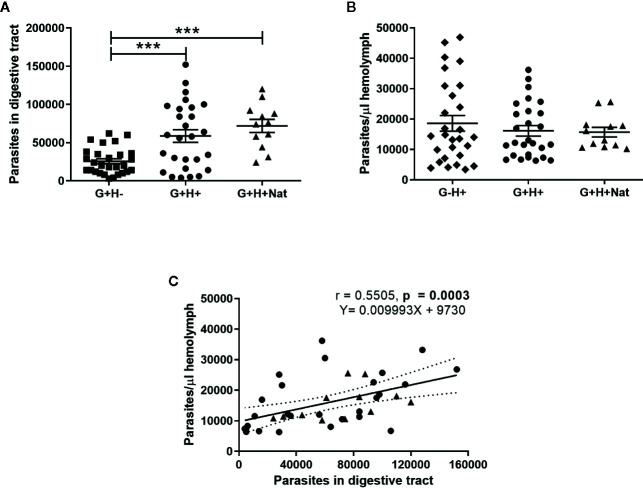
*T. rangeli* loads in the gut and hemolymph of *R. prolixus* with different forms of infection. **(A, B)** Parasite numbers in **(A)** digestive tract and **(B)** hemolymph. Each point represents the numbers of parasites found in individual insects. Error bars represent the mean ± SEM. Asterisks correspond to significant differences (*** p < 0.001) obtained by one-way ANOVA followed by Bonferroni multiple-comparison *post-hoc* test. **(C)** Correlation between *T. rangeli* numbers in gut and hemolymph. Each point represents the numbers of parasites measured in the gut and in the hemolymph of individual insects. The regression line calculated from the data and its 95% confidence intervals are also represented (continuous and curved dotted lines, respectively). G+H−: insects with parasites in the gut, but not in the hemolymph (represented by squares); G−H+: insects that were only infected in the hemolymph by inoculation (represented by diamonds); G+H+: insects with parasites in the gut and further inoculated with parasites in the hemolymph (represented by circles); and G+H+Nat: insects that were only gut-infected, but with parasites in the hemolymph by “natural” crossing from the intestinal lumen (represented by triangles). Each experimental group contained insects from three biological replicates (n = 26–28), with 8–10 insects from each replicate (except for n = 12 in the G+H+Nat group, composed of eight and four insects from two replicates).

We found significant differences in the number of parasites present in the gut of insects that were orally infected but differed in the presence of *T. rangeli* in the hemolymph (**Figure 1A**, ANOVA, p < 0.001). Specifically, we detected lower parasite loads in G+H− insects compared to G+H+ or G+H+Nat (**Figure 1A**, Bonferroni’s *post-hoc* test, p < 0.001 for all mentioned comparisons). Conversely, when we analyzed *T. rangeli* numbers in the hemolymph of inoculated or spontaneous infected insects, we found similar parasite loads, regardless of the presence of *T. rangeli* in the gut (**Figure 1B**, ANOVA, p > 0.05). These results suggest that *T. rangeli* numbers in the digestive tract of *R. prolixus* could be affected by the presence of the parasite in the hemolymph. In line with this, we observed a positive correlation between parasite numbers in the gut and the hemolymph (**Figure 1C**, Spearman r = 0.55, p < 0.001). Indeed, this positive correlation was maintained when we analyzed the G+H+ and G+H+Nat groups of insects separately (**Supplementary Figure 1**), indicating higher parasite loads in the gut of *R. prolixus* when *T. rangeli* numbers are also elevated in the hemolymph.

### Hemolymph Infection Increases *T. rangeli* Numbers in the Gut of Orally Infected *R. prolixus*

**Figure 2:**
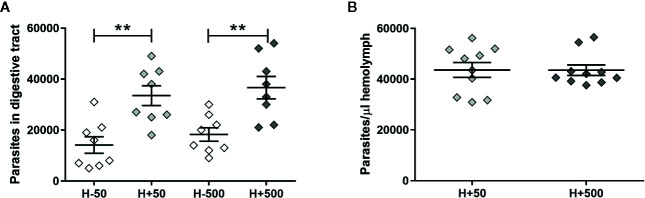
*T. rangeli* loads in the gut and hemolymph of hemolymph-infected *R. prolixus* that were later orally infected. Parasite numbers in **(A)** digestive tract and **(B)** hemolymph. Each point represents the numbers of parasites found in individual insects. Error bars represent the mean ± SEM. Data was analyzed using one-way ANOVA followed by Bonferroni multiple-comparison *post-hoc* test **(A)** or Mann-Whitney U test **(B)**. Asterisks correspond to significant differences (** p < 0.01). H+50: insects infected in the hemolymph by inoculation with 50 epimastigotes; H+500: insects infected in the hemolymph by inoculation with 500 epimastigotes; H−50 and H−500: control groups with PBS + LIT intracoelomic inoculation. Two weeks after the inoculation all groups of insects were orally infected, and parasite counts were determined one week later. Each experimental group contained insects from two biological replicates (n = 8–10), with four to five insects from each replicate.

To further support the hypothesis that the presence of *T. rangeli* in the hemolymph could affect the parasite numbers in the gut, we first inoculated insect hemocoel with PBS + 50 or 500 epimastigotes (H+50 and H+500, respectively) or with PBS + LIT (H−50 and H−500, respectively), and then we proceeded with the oral infection (Experimental design 2 of the Methods section). Afterward, we evaluated the parasite loads in the gut and in the hemolymph of the different experimental groups (**Figure 2**). We evidenced higher parasite numbers in the gut of the H+50 and H+500 groups compared to insects from their respective mock-inoculated control groups (**Figure 2A**, ANOVA, p < 0.001; Bonferroni’s *post-hoc* test, p < 0.01 for H−50 vs. H+50 and for H−500 vs. H+500). Besides, we found no differences in *T. rangeli* loads in the gut between insects that were inoculated with different parasite doses (**Figure 2A**, Bonferroni’s *post-hoc* test, p > 0.05 for H+50 vs. H+500). Moreover, we detected similar numbers of parasites in the hemolymph between insects inoculated with 50 or 500 epimastigotes (**Figure 2B**, Mann-Whitney U test, p > 0.05). These results indicate that the parasite load achieved in the hemolymph was similar regardless of the inoculation dose used, but that *T. rangeli* infection in the hemolymph increased the parasite numbers in the gut of orally infected *R. prolixus*.

### Differential Expression of IMD, Toll, and Jak/STAT Immune Pathway Genes in *R. prolixus* With Different Forms of *T. rangeli* Infection

**Figure 3:**
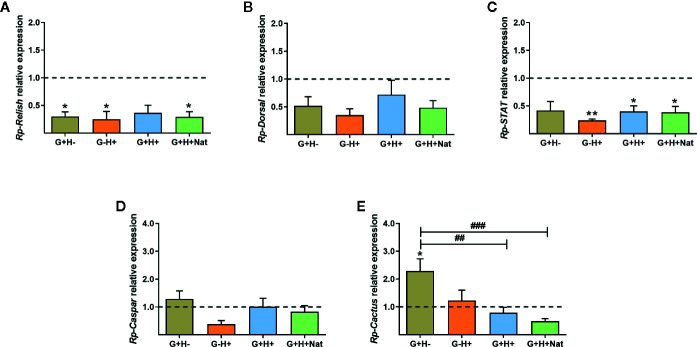
Expression of IMD, Toll, and Jak/STAT immune pathway genes in the fat body of *R. prolixus* with different forms of *T. rangeli* infection. Relative mRNA abundance of the transcription factors **(A)** *Rp-Relish*, **(B)** *Rp-Dorsal*, and **(C)** *Rp-STAT*, and the inhibitors **(D)** *Rp-Caspar* and **(E)** *Rp-Cactus*. All RT-qPCR data were analyzed with the Pfaffl Method, using *α-Actin* as reference gene and normalizing to the gene expression of control insects (represented by the dotted line). Error bars represent the mean ± SEM. Asterisks correspond to significant differences (*p < 0.05 and **p < 0.01) as compared to control, and numerals correspond to significant differences (^##^p < 0.01 and ^###^p < 0.001) between the selected experimental groups, obtained by one-way ANOVA followed by Bonferroni multiple-comparison *post-hoc* test. G+H-: insects with parasites in the gut, but not in the hemolymph; G−H+: insects that were only infected in the hemolymph by inoculation; G+H+: insects with parasites in the gut and further inoculated with parasites in the hemolymph; and G+H+Nat: insects that were only gut-infected, but with parasites in hemolymph by “natural” crossing from the intestinal lumen. Each experimental group contained insects from three biological replicates (n = 8–10), with two to four insects from each replicate (except for two replicates in the G+H+Nat group, composed of four insects each).

As the vector immune response is directly involved in parasite killing and is critical for determining the ability of the parasite to establish the infection, we next investigated whether there were differences in the expression of IMD, Toll, and Jak/STAT immune pathway genes in the fat body of *R. prolixus* with different forms of *T. rangeli* infection. We evidenced that the expression of the TFs *Rp-Relish* and *Rp-STAT* and the inhibitor *Rp-Cactus* differed significantly between the groups of insects analyzed (**Figure 3**, ANOVA, p < 0.01 for the three genes). Particularly related to the IMD pathway, we found the TF *Rp-Relish* downregulated in the G+H−, G−H+, and G+H+Nat groups compared to uninfected insects (**Figure 3A**, Bonferroni’s *post-hoc* test, p < 0.05 for all mentioned comparisons), and similar levels of the inhibitor *Rp-Caspar* between the different experimental groups (**Figure 3D**, ANOVA, p > 0.05). Moreover, regarding the Toll pathway, we measured similar expression of the TF *Rp-Dorsal* between the different groups of insects (**Figure 3B**, ANOVA, p > 0.05). Nevertheless, we evidenced higher levels of the inhibitor *Rp-Cactus* in G+H− insects compared to the G−H− (control insects), G+H+ and G+H+Nat groups (**Figure 3E**, Bonferroni’s *post-hoc* test, p < 0.05 for G+H− vs. G−H−, p < 0.01 for G+H− vs. G+H+ and p < 0.001 for G+H− vs. G+H+Nat). Furthermore, concerning the STAT pathway, we observed a lower expression of the TF *Rp-STAT* in the G−H+, G+H+, and G+H+Nat groups when compared to uninfected insects (**Figure 3C**, Bonferroni’s *post-hoc* test, p < 0.05 for G+H+ vs. G−H− and p < 0.01 for G−H+ vs. G−H− and G+H+Nat vs. G−H−). Overall, these data demonstrate that key components of the IMD, Toll, and Jak/STAT immune pathways are differentially modulated in *R. prolixus* when *T. rangeli* infects the gut, the hemolymph or both regions of the insect vector.

### Correlation Between Parasite Loads and the Expression of Immune Pathway Genes in the Fat Body of *R. prolixus* Infected With *T. rangeli*

**Figure 4:**
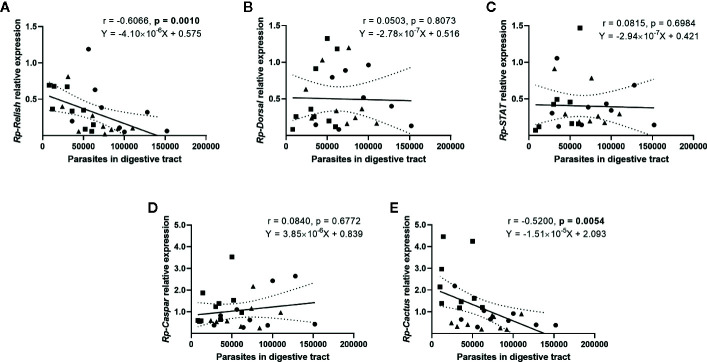
Correlation between the parasite load in the gut and the expression of IMD, Toll, and Jak/STAT immune pathway genes in R*. prolixus* with different forms of *T. rangeli* infection. Correlation between *T. rangeli* numbers in the gut and the relative mRNA abundance of the transcription factors **(A)** *Rp-Relish*, **(B)** *Rp-Dorsal*, and **(C)** *Rp-STAT*, and the inhibitors **(D)** *Rp-Caspar* and **(E)** *Rp-Cactus*. Each point represents the gene expression and the numbers of parasites measured in the fat body of individual insects (n = 25–27), from a total of three biological replicates composed of 8-10 insects each. Squares represent insects with parasites in the gut, but not in the hemolymph (G+H-); circles represent insects with parasites in the gut and further inoculated with parasites in the hemolymph (G+H+); and triangles represent insects that were only gut-infected, but with parasites in hemolymph by "natural" crossing from the intestinal lumen (G+H+Nat). Each graph includes the regression line calculated from the data and its 95% confidence intervals (continuous and curved dotted lines, respectively).

**Figure 5:**
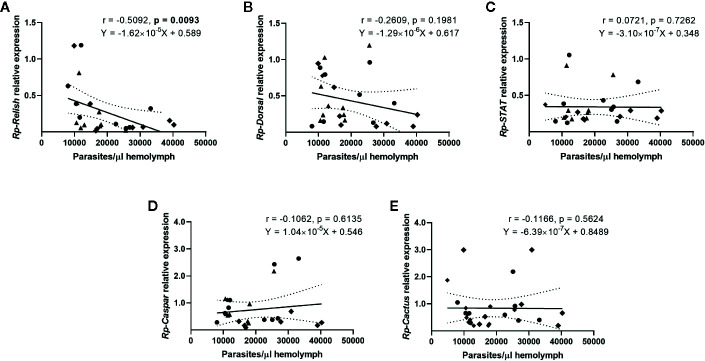
Correlation between the parasite load in the hemolymph and the expression of IMD, Toll, and Jak/STAT immune pathway genes in R*. prolixus* with different forms of *T. rangeli* infection. Correlation between *T. rangeli* numbers in the hemolymph and the relative mRNA abundance of the transcription factors **(A)** *Rp-Relish*, **(B)** *Rp-Dorsal*, and **(C)** *Rp-STAT*, and the inhibitors **(D)** *Rp-Caspar* and **(E)** *Rp-Cactus*. Each point represents the gene expression and the numbers of parasites measured in the fat body of individual insects (n = 25–27), from a total of three biological replicates composed of 8–10 insects each. Diamonds represent insects that were only infected in the hemolymph by inoculation (G-H+); circles represent insects with parasites in the gut and further inoculated with parasites in the hemolymph (G+H+); and triangles represent insects that were only gut-infected, but with parasites in hemolymph by "natural" crossing from the intestinal lumen (G+H+Nat). Each graph includes the regression line calculated from the data and its 95% confidence intervals (continuous and curved dotted lines, respectively).

We next evaluated whether there is a correlation between the expression of the IMD, Toll, and Jak/STAT pathway genes and the parasite counts in the gut (**Figure 4**) and the hemolymph (**Figure 5**) of *T. rangeli*-infected insects (**Supplementary Figures 2–6** for correlations analyzing each group of insects separately). We found no association between *Rp-Dorsal* (**Figure 4B**, Spearman r = 0.05, p > 0.05), *Rp-STAT* (**Figure 4C**, Spearman r = 0.08, p > 0.05) or *Rp-Caspar* (**Figure 4D**, Spearman r = 0.08, p > 0.05) and parasite loads in the gut. Nevertheless, we did find a negative correlation between the number of parasites in that organ and the expression of the TF *Rp-Relish* (**Figure 4A**, Spearman r = −0.61, p = 0.001) and the inhibitor *Rp-Cactus* (**Figure 4E**, Spearman r = −0. 52, p < 0.01). Furthermore, we observed no association between *T. rangeli* numbers in the hemolymph and the expression of *Rp-Dorsal* (**Figure 5B**, Spearman r = −0.26, p > 0.05), *Rp-STAT* (**Figure 5C**, Spearman r = 0.07, p > 0.05), *Rp-Caspar* (**Figure 5D**, Spearman r = −0.11, p > 0.05) or *Rp-Cactus* (**Figure 5E**, Spearman r = −0.11, p > 0.05), while we detected a negative correlation between parasite counts and the expression of *Rp-Relish* in the same samples (**Figure 5A**, Spearman r = −0.51, p < 0.01). Taken together, our results indicate a relationship between *T. rangeli* loads in the gut and the hemolymph of *R. prolixus* with the expression of critical genes that modulate different immune pathways during infection.

## Discussion

Several reports in which *R. prolixus* was infected with *T. rangeli* have revealed that the ability of the parasite to reach the hemolymph, modulate the immune response and establish the infection in the salivary glands is dependent on the parasite strain and developmental form (short vs. long epimastigotes) (Grisard et al., 1999; Machado et al., 2001; Whitten et al., 2001; Whitten et al., 2007; Figueiredo et al., 2008; Ferreira et al., 2010; Cosentino-Gomes et al., 2014; Vieira et al., 2015). In this regard, and taking into consideration that insects are more susceptible to strains isolated from the same geographic region, we used for our experiments the CHOACHI strain of *T. rangeli*, which was isolated from naturally infected *R. prolixus* in Colombia and is able to complete its lifecycle inside the vector (Machado et al., 2001; Ferreira et al., 2010). Furthermore, to simulate the natural infection with the parasite, we used *T. rangeli*-infected mice to perform gut infections, and a low dose (50 epimastigotes) of cultured parasites for intracoelomic infections, which were previously passed through triatomines and mice to maintain the strain infectivity. We also discriminated and analyzed a group of insects that were only gut-infected and presented hemolymph infection by spontaneous crossing of parasites from the intestinal lumen (G+H+Nat group), which implies a more physiological approach for our experiments. Correspondingly, and in agreement with a previous report (Ferreira et al., 2010), we found similar results between insects that were orally infected and later inoculated with parasites in the hemolymph (G+H+) and the G+H+Nat group, suggesting that our protocol could be used to mimic the effects of the *T. rangeli* in the natural course of infection, at least during the evaluated period.

Independently of the way insects were infected, we measured similar numbers of parasites in the hemolymph of individuals with systemic infection, indicating that parasite loads in the caelomatic cavity were not affected by the presence of *T. rangeli* in the gut during long-term infections. Comparable numbers of parasites in the hemolymph were found in other studies using an initial dose of 10 or 100 parasites, which showed that the load of *T. rangeli* reaches a plateau of about 10^7^–10^8^ parasites/ml, suggesting that similar results could be achieved after 2 weeks of intracoelomic infection by the inoculation of 10, 50, 100, or 500 parasites (Mello et al., 1995; Ferreira et al., 2010). In addition, and similar to a previous study using the CHOACHI strain, we detected high parasite numbers in the gut of insects 4 weeks after they fed on infected mice (Ferreira et al., 2018). Nevertheless, infections of *R. prolixus* with culture epimastigotes from Macias and H14 strains showed that, during short-term infections, the parasite predominantly colonized the anterior midgut of the bug and its numbers decreased over time, while in long-term infections and after the bug’s molting, the parasite infected the posterior midgut (Gomes et al., 2003; Vieira et al., 2015). Although we dissected the entire digestive tract, the parasite numbers we counted were higher than in those last reports. These discrepancies could be related to differences in infection’s methodology, since the mentioned studies were performed using artificial blood feeding and different *T. rangeli* strains that were maintained exclusively in culture, which may impact parasite infectivity.

Strikingly, we found significantly higher parasite loads in the gut of insects that also had hemolymph infection, in comparison with insects without systemic infection, suggesting that the presence of *T. rangeli* in the hemolymph could influence parasite numbers in the gut during long-term infections. In line with this, we showed a positive correlation between the number of parasites in the hemolymph and in the gut. To confirm the hypothesis that the presence of parasites in the hemolymph could modulate the size of the population in the gut, we inoculated the insects’ hemocoel with parasites and then performed the oral infection. Through this approach, we observed higher parasite loads in the gut of insects with hemolymph infection compared to the PBS + LIT inoculated groups. Overall, we evidenced for the first time that the presence of *T. rangeli* in the hemolymph affects the infection of the digestive tract, enhancing parasite numbers. Thus, we support the notion that this has significance on the course of natural infections and reinfections.

During an infection, the insect immune system recognizes pathogens’ antigens as non-self, initiating several responses against the microbe, which are mainly triggered by the Toll, IMD and Jak/STAT immune pathways (Salcedo-Porras and Lowenberger, 2019). Several reports in different insect species have shown that the presence of parasites in the digestive tract stimulates the production of AMPs by the fat body and their systemic secretion in the hemolymph, even without the invasion into the hemocoel (Boulanger et al., 2001; Hao et al., 2001; Boulanger et al., 2002; Boulanger et al., 2004). Afterward, AMPs can diffuse throughout the insect body, establishing a systemic response that seems to diminish parasite loads in the digestive tract in response to oral infection (Boulanger et al., 2004). The opposite seems to occur in the case of *T. rangeli* infection in triatomines. The oral infection of *R. prolixus* with *T. rangeli* impacts the systemic immune responses by suppressing the prophenoloxidase activating pathway in the hemolymph, and inhibiting microaggregation reactions and phagocytic activity of hemocytes, which apparently facilitates parasite growth in the hemolymph (Gomes et al., 2003; Garcia et al., 2004b; Figueiredo et al., 2008). Besides, *T. rangeli* infection also modulates differentially the production of AMPs and nitric oxide in the gut and in the fat body of *R. prolixus*, varying over time as parasites multiply and the infection is established (Whitten et al., 2007; Vieira et al., 2015). Thus, in this study, we hypothesized that there are differences in the systemic modulation of immune pathways in *R. prolixus* during a long-term infection with *T. rangeli* in the gut or in the hemolymph, and these differences could influence in the number of parasites that survive in both regions of the triatomine body.

There are few studies on the immune pathways elicited in *R. prolixus* during pathogen infection, and it is assumed that these pathways function as in other model insects (Mesquita et al., 2015; Zumaya-Estrada et al., 2018; Salcedo-Porras and Lowenberger, 2019). Recently, the NF-κB TF *Rp-Relish* of the IMD pathway was found to differentially regulate the expression of AMPs in response to Gram-negative and Gram-positive bacteria (Salcedo-Porras et al., 2019). In addition, when *Rp-Relish* expression was silenced, the population of the symbiotic bacteria *Rhodococcus rhodnii* increased in the insect gut (Mesquita et al., 2015). However, it was reported that silencing *Rp-Relish* or the NF-κB TF *Rp-Dorsal* of the Toll pathway did not influence in *T. cruzi* loads in *R. prolixus* gut (Mesquita et al., 2015). Conversely, *T. cruzi* and *T. rangeli* infections modulate the expression of AMPs that are thought to be regulated by the Toll and IMD pathways, indicating that these pathways are functional during parasitic infections (Vieira et al., 2015; Vieira et al., 2016). In this regard, our results suggest a decreased activity of the IMD pathway in *R. prolixus* during long-term infections with *T. rangeli*. Accordingly, we demonstrated a ~70%–75% downregulation of the TF *Rp-Relish* and no changes in the expression of the inhibitor *Rp-Caspar* of the IMD pathway in insects with oral and/or systemic infection, in comparison with uninfected controls. Besides, we found an inverse correlation between the numbers of parasites in the gut and the expression of *Rp-Relish*, suggesting that higher numbers of *T. rangeli* in the digestive tract could increase the systemic repression of the IMD pathway in orally infected *R. prolixus*. Moreover, we detected an inverse correlation between parasite counts in the hemolymph and the expression of this TF, pointing that the activation of the IMD pathway could be reduced by higher parasite loads in the hemolymph of insects with systemic infection.

Regarding the Toll pathway, we found no differences in *Rp-Dorsal* expression between the groups of insects analyzed, but a ~2- to 4-fold higher levels of the inhibitor *Rp-Cactus* in the G+H− group, compared to controls and insects with both oral and systemic infection, suggesting a downregulation of this pathway in gut-only infected insects, which could be reverted when *T. rangeli* also infects the hemolymph. This differential expression of *Rp-Cactus* between the G+H− compared to the G+H+ and G+H+Nat groups of insects could be related to the differences in parasite loads found between insect with or without hemolymph infection. Accordingly, we found a negative correlation between *Rp-Cactus* expression and parasite loads in the gut, indicating that when there are low parasite counts in the gut, the expression of *Rp-Cactus* is high (as in the G+H− group); or when the parasite numbers in the gut are high, the expression of *Rp-Cactus* is lower (as in G+H+ and G+H+Nat groups). Supporting our findings, another study demonstrated that the phenoloxidase activity and *Rp-Prolixicin*, *Rp-Lysozyme A*, *Rp-Lysozyme B*, and *Rp-Defensin B* gene expression (AMPs induced by the activation of immune pathways) are suppressed in the digestive tract of *R. prolixus* after oral infection with this parasite, indicating a systemic down-regulation of the immune pathways by *T. rangeli* infection (Vieira et al., 2015). In addition, studies on *Anopheles gambiae*, *Glossina morsitans*, *Phlebotomus papatasi*, and *Aedes aegypti* infected with different parasites (*Plasmodium* sp., *Trypanosoma* sp. and *Leishmania* sp.) showed that the expression of *Relish* and *Cactus* regulates infection prevalence and intensity, highlighting the importance of the IMD and Toll pathways in anti-parasitic immune responses in vectors (Meister et al., 2005; Frolet et al., 2006; Hu and Aksoy, 2006; Antonova et al., 2009; Louradour et al., 2019). In this regard, a recent report demonstrated a reduction in *T. cruzi* loads in the midgut of *R. prolixus* treated with the drug IMD-0354, which impedes the activation of NF-κB TFs (Vieira C. S. et al., 2018).

The TF *Rp-STAT* was first sequenced in transcriptome analyses from the digestive tract of non-infected *R. prolixus* (Ribeiro et al., 2014). Afterward, several members of the Jak/STAT pathway were identified in transcriptome analyses of *Triatoma infestans*, *Triatoma dimidiata*, and *Triatoma pallidipennis*, and in the genome of *R. prolixus*, suggesting that this pathway is functional in triatomines (Mesquita et al., 2015; Zumaya-Estrada et al., 2018). Nevertheless, as far as we know, the expression and function of this pathway in triatomines during microbial infection has not been studied. In this work, we did not find any correlation between parasite loads and the expression of *Rp-STAT*, suggesting that the Jak/STAT pathway is not modulated by *T. rangeli* numbers in *R. prolixus*. Strikingly, in comparison with uninfected insects, we determined a ~60%–75% lower expression of *Rp-STAT* in all groups of insects with hemolymph infection, suggesting that *T. rangeli* reduces the Jak/STAT pathway activity when infecting the hemocoel, independently of the presence of parasites in the gut. This downregulation of *Rp-STAT* could be needed for the parasite to proliferate and increase its number in the hemolymph after the hemocoel infection. Besides, it could also be related to the higher *T. rangeli* numbers found in the gut of hemolymph infected insects, as the systemic repression of the Jak/STAT pathway could influence in the parasite loads in the gut. In agreement with this hypothesis, STAT knockdown by RNAi increased the number of *Plasmodium vivax* in the midgut of *Anopheles aquasalis*, and enhanced *Plasmodium berghei* and *Plasmodium falciparum* infection in the digestive tract of *A. gambiae* (Gupta et al., 2009; Bahia et al., 2011). Further studies are needed to also confirm the anti-parasitic role of this pathway during *T. rangeli* and *R. prolixus* interactions.

It is worth mentioning that, to date, no study has examined whether IMD, Toll, and Jak/STAT immune pathway genes are modulated in *R. prolixus* by *T. rangeli* infection. Nevertheless, we are aware that gene expression of regulatory factors in immune pathways does not necessarily reflect the activation of these pathways, as they are often regulated by post-transcriptional modifications. However, to our knowledge, specific antibodies to detect the cellular localization of these proteins or the phosphorylation status are not available yet for *R. prolixus*. Furthermore, a larger-scale gene expression analysis will be important to reveal whether there is a global modulation in each of the immune pathways’ genes (receptors, signaling cascade components) besides their TFs/inhibitors. On the other hand, compared to *Drosophila melanogaster*, in which the expression of certain AMPs (i.e. the AMP *Drosomycin* for the Toll pathway or the AMP *Diptericin* for the IMD pathway) directly reflects the activity of specific immune pathways, the specificity of AMPs expression triggered by each of the immune pathways are unknown in *R. prolixus* (Lemaitre et al., 1997; Khush et al., 2001; Michel et al., 2001; Gottar et al., 2002; Rutschmann et al., 2002; Tauszig-Delamasure et al., 2002; Hanson et al., 2019; Lin et al., 2019). In fact, to date only two studies reported modulation of AMPs after silencing *Rp-Relish*, with discrepancies between them (Mesquita et al., 2015; Salcedo-Porras et al., 2019). In addition, there is no information regarding AMPs expression after silencing genes of the Toll or Jak/STAT pathway in *R. prolixus*, which does not allow the comparison with the mentioned reports on the IMD pathway. Consequently, we considered that measuring the expression of TFs and negative regulators that are specific for each of the immune pathways would be a feasible way to evaluate how these pathways are modulated in *R. prolixus*.

The aim of this work was to analyze the systemic modulation of immune pathways in *R. prolixus* during long-term infection with *T. rangeli* as a mean to uncover which immune pathways play a role during the different phases of the infection in the insect. Accordingly, we demonstrated a relationship between parasite counts in the digestive tract/hemolymph and gene expression in the fat body, but local immune responses in the digestive tract of triatomines or triggered by hemocytes also play important roles in controlling microbial infections at different sites (Mello et al., 1995; Whitten et al., 2001; de Oliveira and de Souza, 2003; Garcia et al., 2004a; Castro et al., 2012; Vieira et al., 2014; Mesquita et al., 2015; Vieira et al., 2016; Favila-Ruiz et al., 2018; Vieira C. S. et al., 2018). In fact, it was shown that oral infection with *T. rangeli* modulates AMPs and NOS expression in the gut of *R. prolixus*; and systemic infections increase hemocyte numbers, hemocyte microaggregation, ROS and NOS levels in the hemolymph, and the activity of the prophenoloxidase (proPO) system (Whitten et al., 2001; Garcia et al., 2004a; Whitten et al., 2007; Vieira et al., 2015). Thus, additional studies analyzing which immune pathways are modulated in the digestive tract/hemocytes of *R. prolixus* during *T. rangeli* infection and whether there is an association between these pathways and parasite loads will yield valuable information to complement the present and mentioned reports.

It should be noted that in the present study we analyzed individual insects, and thus inherent high variability between samples are expected for parasite loads and gene expression. In line with this, we consider that the main source of variation came from the inter-individual genetic differences in the population of parasites and insects. Although we worked with only one strain of *T. rangeli* (CHOACHI) and a single colony of *R. prolixus*, the individuals forming those populations are not genetic clones. Thus, while all insects were infected at the same time and under similar conditions, the numbers of parasites infecting each individual, the immune responses elicited, and the course of the infection will depend on the specific interactions between the insects and the parasites infecting it. In this regard, previous reports analyzing individual insects using the same colony of *R. prolixus* and *T. rangeli* strain have demonstrated this variation (Ferreira et al., 2010; Ferreira et al., 2015; Ferreira et al., 2018). In addition, during the oral feeding, we allowed the insects to ingest 20–30 μl of blood, which resulted in ingestion of approximately 50–150 trypomastigotes for the infections. These differences in the initial dose of ingested parasites per insect may also contribute to the variance observed in our results. Another source of variation came from the spontaneous infection of the hemolymph in orally infected insects, as we cannot control whether or when the parasite crosses the gut epithelium to the hemocoel. Thus, although we were able to discriminate insects with spontaneous infection in the hemolymph (G+H+Nat group), probably the passage from the gut occurred at different time in different insects, influencing in the results obtained within this group of insects and between other groups. Finally, for the case of the association found between parasite loads and gene expression, the data correspond to individual insects infected in the gut or the hemolymph, independently of other types of infection. Hence, it is likely that whether insects are infected in other sites would influence in the results of the correlations. Accordingly, we showed the correlation for all groups of insects separately in **Supplementary Figures 2–6**. However, in this latter analysis, the numbers of individual drop considerably in each correlation (<10 insects per group), and we believe that the information of more insects is needed to get robust conclusions from these results.

Another limitation of this work is that only one timepoint was chosen for the experiments. *Trypanosoma rangeli* is a parasite that produce chronic systemic infections in *Rhodnius* (colonizing the gut, hemolymph and salivary gland of the insect) (Guarneri and Lorenzo, 2017). It is unclear how and when *T. rangeli* spontaneously cross the gut epithelium and colonizes the hemolymph; and it is a process that occurs in a very low percentage (2%–13%) of orally infected insects (Hecker et al., 1990; de Oliveira and de Souza, 2001; Ferreira et al., 2010; Ferreira et al., 2015). When hemolymph invasion happens, the development of the parasite in the insect is usually slow, as it takes approximately 3 weeks after entering in the hemolymph to produce metacyclic forms in the salivary glands (Ferreira et al., 2010; Paim et al., 2013). Therefore, we chose the period of 4 weeks to make our measurements because we wanted that all developmental forms were established. However, future research with a temporal kinetics will be very interesting to complement our findings, as it would uncover how the immune pathways are modulated by parasite loads during the entire infection and how the dynamics in parasite loads in the gut or hemolymph influence in the number of *T. rangeli* presents in other regions of the insect body, shedding more light into the communication between the gut and the hemolymph along the course of the infection. Thus, even though we could not show the modulation of the immune system over time, our results revealed important features of the *T. rangeli-R. prolixus* interaction. The fact that the parasite can sustain distinct immunomodulation in long-term infections, depending on the site of infection, indicates that these differences are crucial to complete its entire biological cycle inside the insect. Moreover, taking into consideration that most of the infected insects will not develop a systemic infection, the fact that gut only-infected bugs maintain two immune activation pathways reduced (Toll and IMD, as suggested by our results) in a long-term infection may be important for the maintenance of stable populations with a high number of parasites in the intestinal tract, which could increase the chances of hemolymph invasion (Hecker et al., 1990; de Oliveira and de Souza, 2001; Ferreira et al., 2010; Ferreira et al., 2015; Ferreira et al., 2018). Similarly, the sustained repression of two immune pathways (IMD and Jak/STAT) in hemolymph-infected *R. prolixus* during long-term infection may be responsible for the high parasite loads found in that region of the insect body, which could facilitate the infection of the salivary glands (Mello et al., 1995; Ferreira et al., 2010).

**Figure 6:**
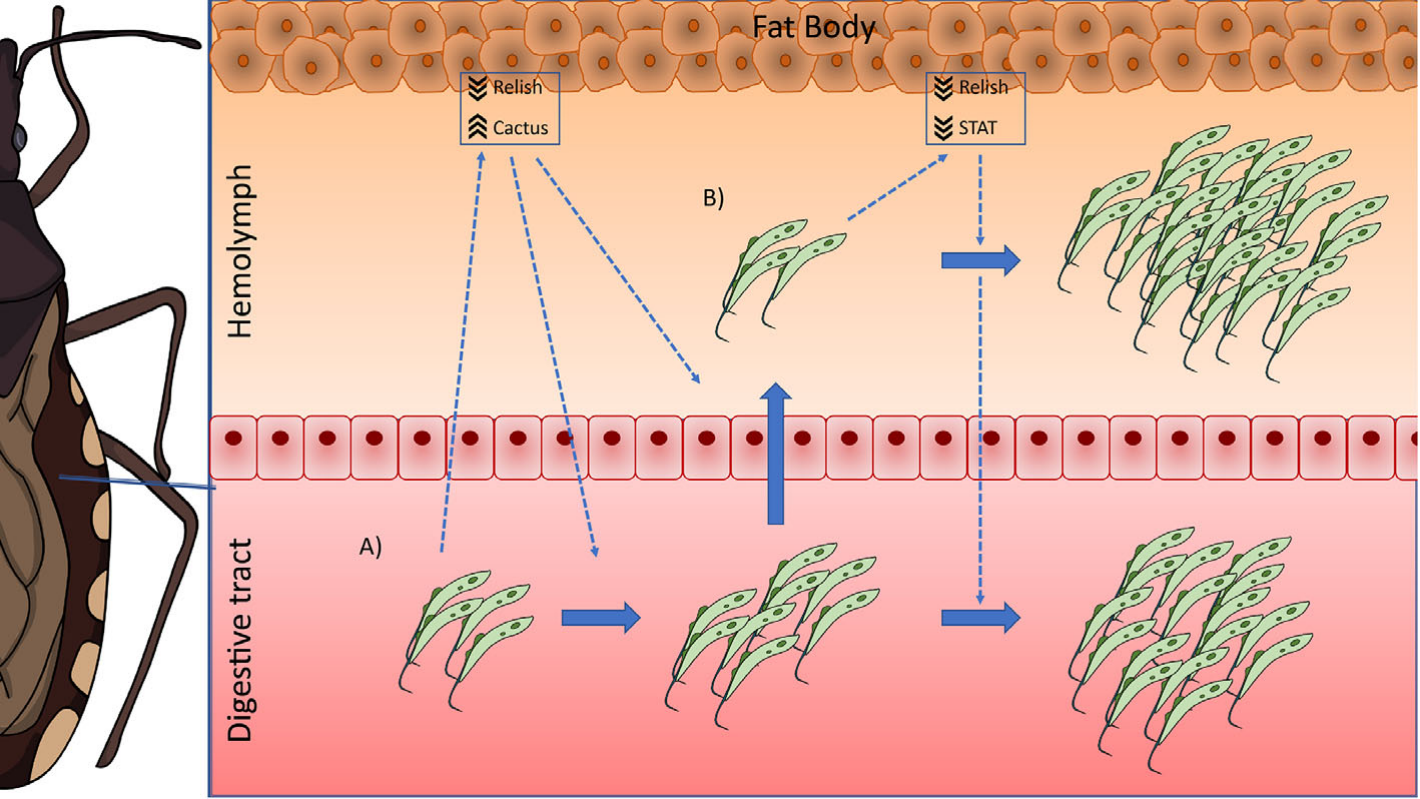
IMD, Toll, and Jak/STAT immune pathway genes modulation in *R. prolixus* during long-term infection with *T. rangeli*. Possible scenario: **(A)** When *T. rangeli* infects the digestive tract of *R. prolixus*, it causes a systemic decrease in the activation of the Toll (by upregulating the levels of the inhibitor *Rp-Cactus*) and IMD (by downregulating the TF *Rp-Relish*) immune pathways, which allows the setting of the infection. This modulation of the immune response would also trigger the multiplication of the first parasites that reach the hemocoel, which proliferate and establish the infection in the hemolymph. **(B)** During hemolymph infection, the systemic inhibition of the IMD pathway remains (downregulation of *Rp-Relish*) but not the repression in the Toll pathway (*Rp-Cactus* levels return to basal); and the systemic activity of the Jak/STAT immune pathway is now also diminished (downregulation of the TF *Rp-STAT*). In consequence, parasite loads in the hemolymph and the digestive tract increase.

Based on our results, we postulate the following possible scenario in *R. prolixus* during long-term infections with *T. rangeli*: when the parasite infects the digestive tract of the insect, it causes a systemic decrease in the activation of the Toll (by upregulating the levels of the inhibitor *Rp-Cactus*) and IMD (by downregulating the TF *Rp-Relish*) immune pathways, which allows the setting of the infection. This modulation of the immune response would also trigger the multiplication of the first parasites that reach the hemocoel, which proliferate and establish the infection in the hemolymph. During that process, the systemic inhibition of the IMD pathway remains (downregulation of *Rp-Relish*) but not the repression in the Toll pathway (*Rp-Cactus* levels return to basal); and the systemic activity of the Jak/STAT immune pathway is now also diminished (downregulation of the TF *Rp-STAT*). In consequence, parasite loads in the hemolymph and digestive tract increase (**Figure 6**). Our studies open new and challenging questions: Which are the effector mechanisms triggered by each immune pathway to modify *T. rangeli* loads? Is the downregulation of the Jak/STAT pathway necessary for the parasite to invade the salivary glands? Can we inhibit the parasite to infect the hemolymph after an oral infection by upregulating the activation of these immune pathways? RNAi/CRISPR experiments targeting the transcription factors and inhibitors of the IMD, Toll, and Jak/STAT pathways will follow suit to understand the immunological processes involved in *T. rangeli*-*R. prolixus* interaction.

## Data Availability Statement

The original contributions presented in the study are included in the article/supplementary material. Further inquiries can be directed to the corresponding authors.

## Ethics Statement

The animal study was reviewed and approved by the Ethics Committee in Animal Experimentation (CEUA/FIOCRUZ) under the protocol number LW-8/17. This protocol adheres to the guidelines of National Council for Animal Experimentation Control (CONCEA/MCT), which is the maximum ethics committee of the Brazilian government.

## Author Contributions

AR and AG designed the study. AR, AN, and LS were in charge of insect rearing, trypanosome culture, insect infections, and dissections. AR performed the quantification of parasite loads and qRT-PCRs. AR and AG analyzed the data and wrote the original draft of the manuscript. RR-P reviewed and edited the manuscript. All authors contributed to the article and approved the submitted version.

## Funding

This work was supported by project grants from Universidad Nacional del Noroeste de la Provincia de Buenos Aires (SIB 0615/2019 to AR), Roemmers Foundation (FAJR 2018 to AR), Agencia Nacional de Promoción de Ciencia y Tecnología (PICT-2014-1554 to RR-P), and by travel grants from Universidad Nacional del Noroeste de la Provincia de Buenos Aires (SIDT N° 402/2018 and N° 458/2019 to AR). This work was also supported by Fundação de Amparo à Pesquisa do Estado de Minas Gerais (FAPEMIG, grant numbers CRA-APQ-00569-15 and CRA-PPM-00162-17), Instituto Nacional de Ciência e Tecnologia em Entomologia Molecular (INCTEM/CNPq, grant number 465678/2014-9). This study was financed in part by the Coordenação de Aperfeiçoamento de Pessoal de Nível Superior – Brasil (CAPES) – Finance Code 001.

## Conflict of Interest

The authors declare that the research was conducted in the absence of any commercial or financial relationships that could be construed as a potential conflict of interest.
