# Supplementary material for: Modulation of IMD, Toll, and Jak/STAT Immune Pathways Genes in the Fat Body of Rhodnius prolixus During Trypanosoma rangeli Infection
Source: Front Cell Infect Microbiol. 2021 Jan 18;10:598526. doi: 10.3389/fcimb.2020.598526 (PMC7848085; doi:10.3389/fcimb.2020.598526)
Supplement: Supplementary file 1 [file DataSheet_1.docx]

Supplementary Material

**
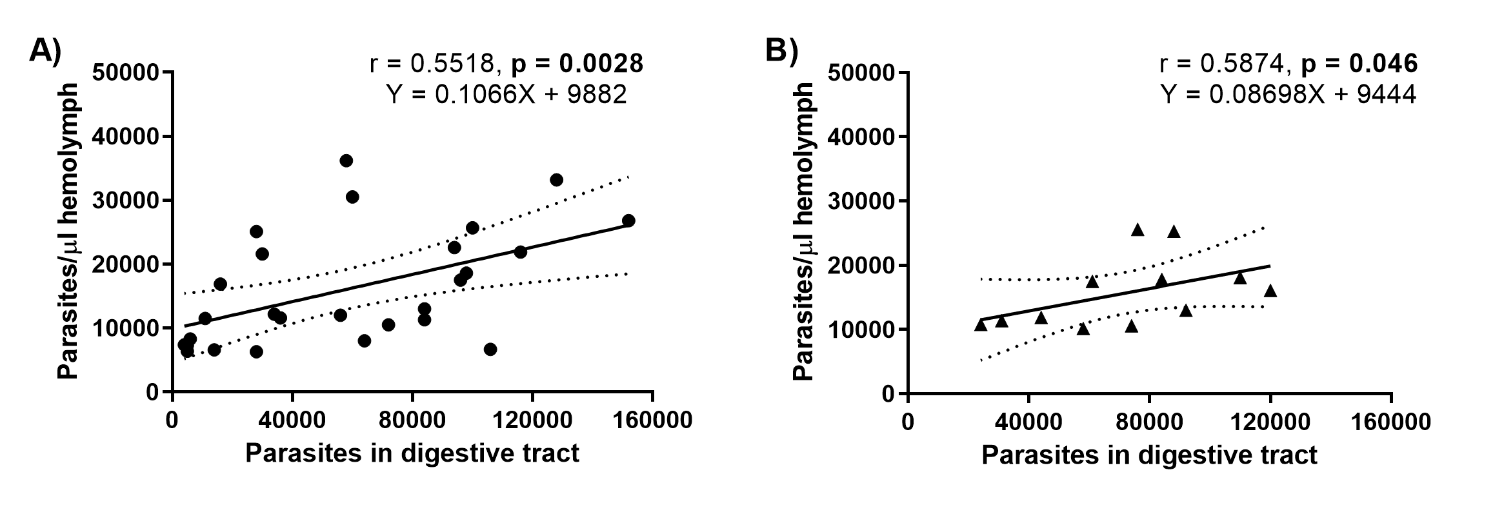
**

**Supplementary Figure 1. Correlation between the parasite load in the gut and hemolymph of *R. prolixus* with different forms of *T. rangeli* infection.** Correlation between *T. rangeli* numbers in the gut and hemolymph of *R. prolixus* from the **(A)** G+H+ (circles) and **(B)** G+H+Nat (triangles) groups of insects. G+H+: insect with parasites in their gut and further inoculated with parasites in the hemolymph; and G+H+Nat: insects that were only gut-infected, but with parasites in hemolymph by "natural" crossing from the intestinal lumen. Each point represents the numbers of parasites measured in the gut and hemolymph of individual insects, from a total of two (G+H+Nat) or three (G+H+) biological replicates. Each graph includes the regression line calculated from the data and its 95% confidence intervals (continuous and curved dotted lines, respectively).


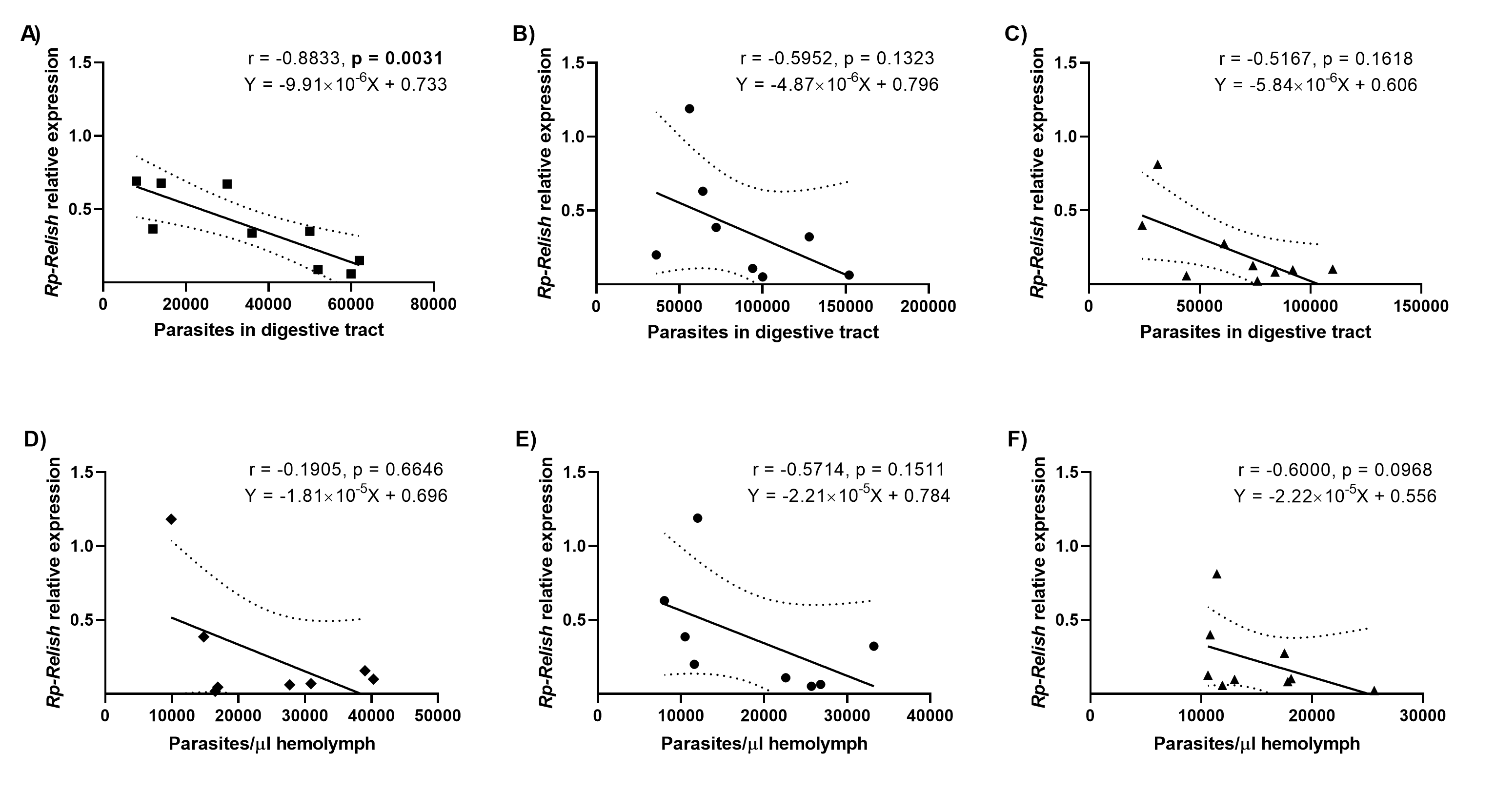


**Supplementary Figure 2. Correlation between the parasite load in the gut or the hemolymph and the expression of the IMD TF *Rp-Relish* in R*. prolixus* with different forms of *T. rangeli* infection.** Correlation between *T. rangeli* numbers in the gut **(A-C)** or the hemolymph **(D-F)** and the relative mRNA abundance of *Rp-Relish* in insect with different forms of infection. G+H-: insects with parasites in the gut, but not in the hemolymph (represented by squares); G-H+: insects that were only infected in the hemolymph by inoculation (represented by diamonds); G+H+: insects with parasites in the gut and further inoculated with parasites in the hemolymph (represented by circles); and G+H+Nat: insects that were only gut-infected, but with parasites in hemolymph by "natural" crossing from the intestinal lumen (represented by triangles). Each point represents the gene expression and the numbers of parasites measured in the gut or hemolymph of individual insects, from a total of three independent experiments. Each graph includes the regression line calculated from the data and its 95% confidence intervals (continuous and curved dotted lines, respectively).


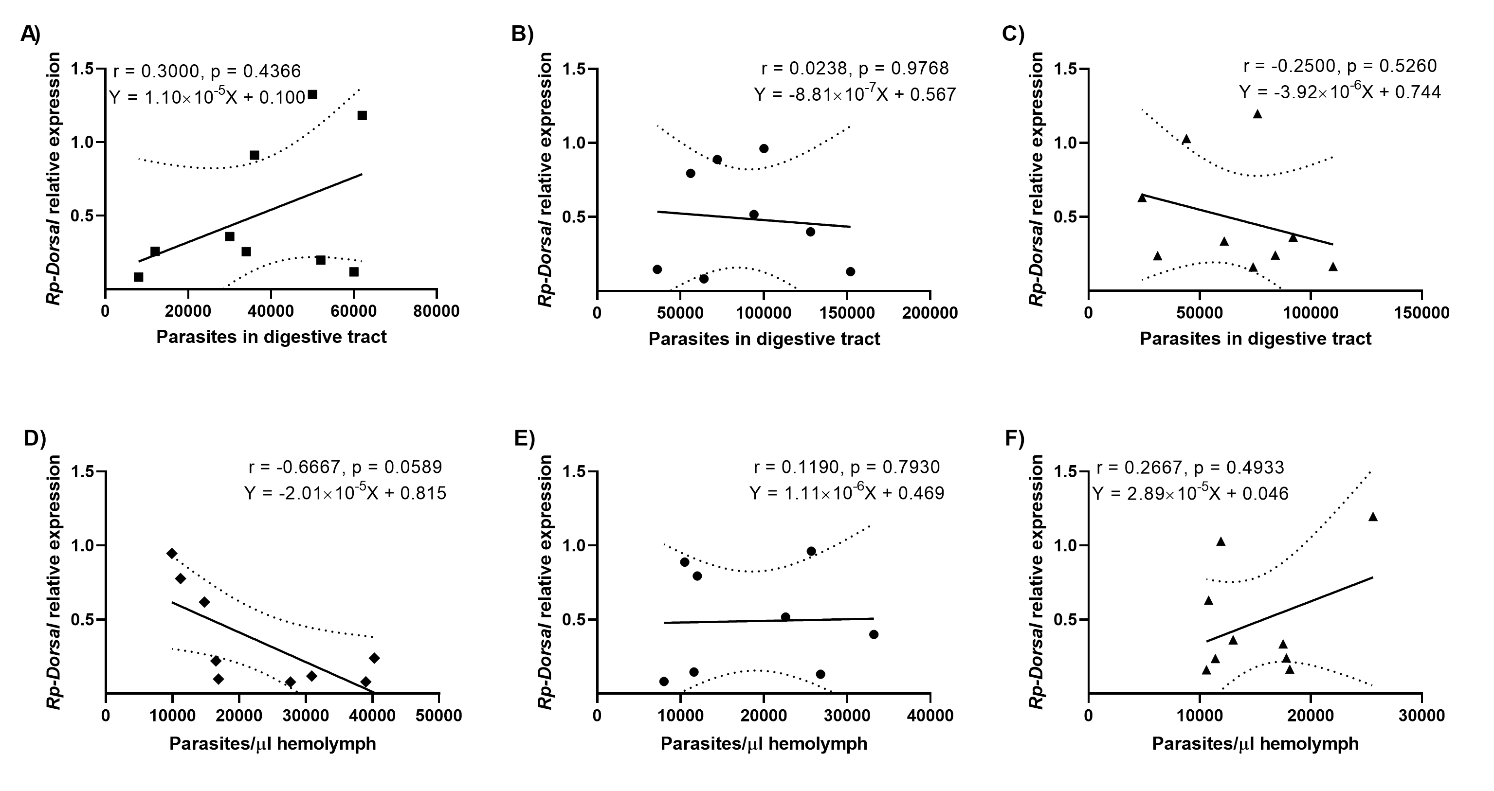


**Supplementary Figure 3. Correlation between the parasite load in the gut or the hemolymph and the expression of the Toll TF *Rp-Dorsal* in R*. prolixus* with different forms of *T. rangeli* infection.** Correlation between *T. rangeli* numbers in the gut **(A-C)** or the hemolymph **(D-F)** and the relative mRNA abundance of *Rp-Dorsal* in insect with different forms of infection. G+H-: insects with parasites in the gut, but not in the hemolymph (represented by squares); G-H+: insects that were only infected in the hemolymph by inoculation (represented by diamonds); G+H+: insects with parasites in the gut and further inoculated with parasites in the hemolymph (represented by circles); and G+H+Nat: insects that were only gut-infected, but with parasites in hemolymph by "natural" crossing from the intestinal lumen (represented by triangles). Each point represents the gene expression and the numbers of parasites measured in the gut or hemolymph of individual insects, from a total of three independent experiments. Each graph includes the regression line calculated from the data and its 95% confidence intervals (continuous and curved dotted lines, respectively).


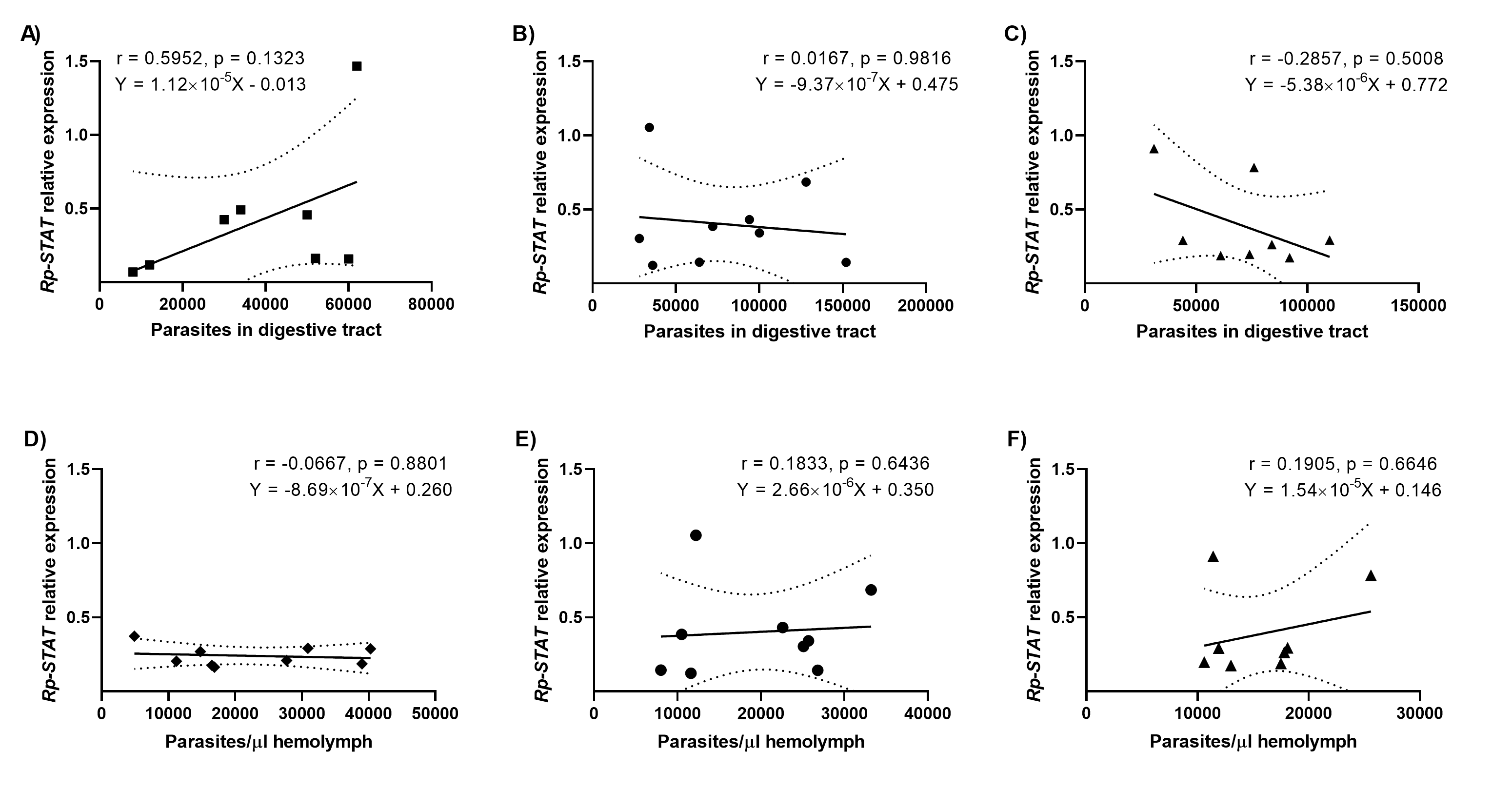


**Supplementary Figure 4. Correlation between the parasite load in the gut or the hemolymph and the expression of the Jak/STAT TF *Rp-STAT* in R*. prolixus* with different forms of *T. rangeli* infection.** Correlation between *T. rangeli* numbers in the gut **(A-C)** or the hemolymph **(D-F)** and the relative mRNA abundance of *Rp-STAT* in insect with different forms of infection. G+H-: insects with parasites in the gut, but not in the hemolymph (represented by squares); G-H+: insects that were only infected in the hemolymph by inoculation (represented by diamonds); G+H+: insects with parasites in the gut and further inoculated with parasites in the hemolymph (represented by circles); and G+H+Nat: insects that were only gut-infected, but with parasites in hemolymph by "natural" crossing from the intestinal lumen (represented by triangles). Each point represents the gene expression and the numbers of parasites measured in the gut or hemolymph of individual insects, from a total of three independent experiments. Each graph includes the regression line calculated from the data and its 95% confidence intervals (continuous and curved dotted lines, respectively).


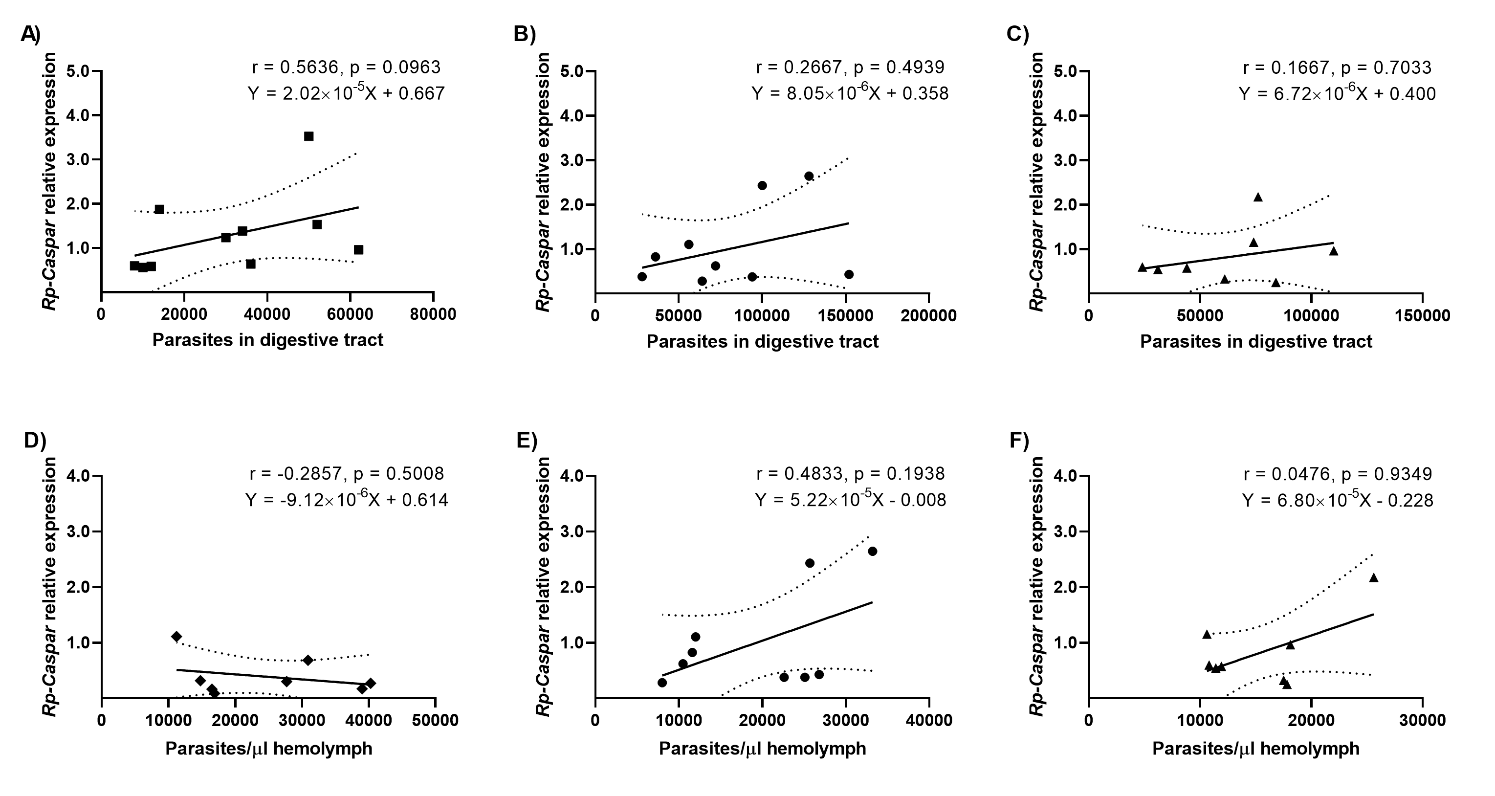


**Supplementary Figure 5. Correlation between the parasite load in the gut or the hemolymph and the expression of the IMD inhibitor *Rp-Caspar* in R*. prolixus* with different forms of *T. rangeli* infection.** Correlation between *T. rangeli* numbers in the gut **(A-C)** or the hemolymph **(D-F)** and the relative mRNA abundance of *Rp-Caspar* in insect with different forms of infection. G+H-: insects with parasites in the gut, but not in the hemolymph (represented by squares); G-H+: insects that were only infected in the hemolymph by inoculation (represented by diamonds); G+H+: insects with parasites in the gut and further inoculated with parasites in the hemolymph (represented by circles); and G+H+Nat: insects that were only gut-infected, but with parasites in hemolymph by "natural" crossing from the intestinal lumen (represented by triangles). Each point represents the gene expression and the numbers of parasites measured in the gut or hemolymph of individual insects, from a total of three independent experiments. Each graph includes the regression line calculated from the data and its 95% confidence intervals (continuous and curved dotted lines, respectively).


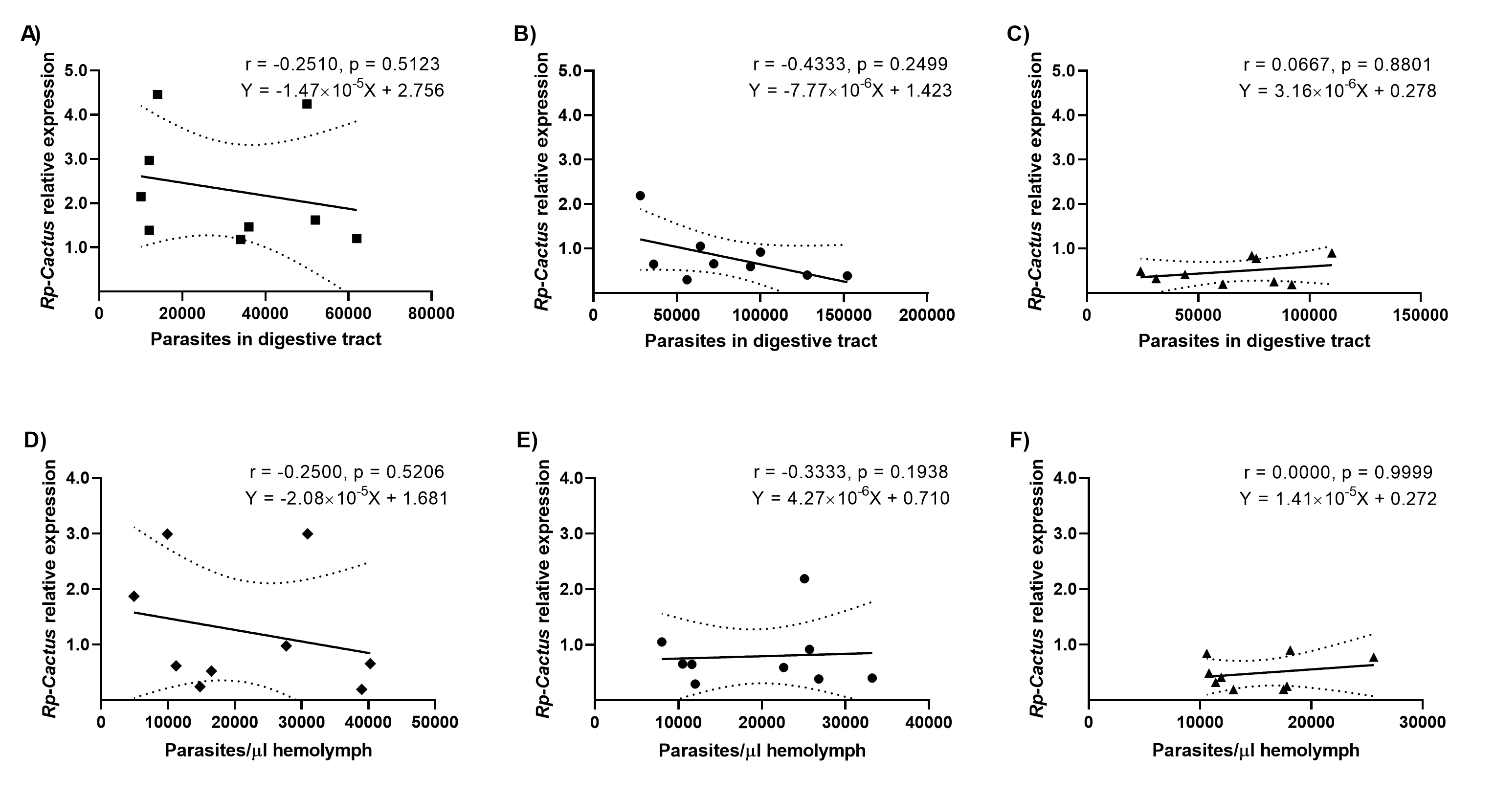


**Supplementary Figure 6. Correlation between the parasite load in the gut or the hemolymph and the expression of the Toll inhibitor *Rp-Cactus* in R*. prolixus* with different forms of *T. rangeli* infection.** Correlation between *T. rangeli* numbers in the gut **(A-C)** or the hemolymph **(D-F)** and the relative mRNA abundance of *Rp-Cactus* in insect with different forms of infection. G+H-: insects with parasites in the gut, but not in the hemolymph (represented by squares); G-H+: insects that were only infected in the hemolymph by inoculation (represented by diamonds); G+H+: insects with parasites in the gut and further inoculated with parasites in the hemolymph (represented by circles); and G+H+Nat: insects that were only gut-infected, but with parasites in hemolymph by "natural" crossing from the intestinal lumen (represented by triangles). Each point represents the gene expression and the numbers of parasites measured in the gut or hemolymph of individual insects, from a total of three independent experiments. Each graph includes the regression line calculated from the data and its 95% confidence intervals (continuous and curved dotted lines, respectively).
